# Attitudes towards community gambling venues and support for regulatory reform: an online panel study of residents in New South Wales, Australia

**DOI:** 10.1186/s12954-018-0218-x

**Published:** 2018-04-02

**Authors:** Amy Bestman, Samantha L. Thomas, Melanie Randle, Hannah Pitt, Mike Daube

**Affiliations:** 1 Centre for Population Health Research, School of Health and Social Development, Faculty of Health, Deakin University, Geelong, Australia; 2 School of Management, Operations and Marketing, Faculty of Business, University of Wollongong, Wollongong, Australia; 3 Faculty of Health Sciences, Curtin University, Perth, Australia

**Keywords:** Gambling, Public health, Electronic gambling machine, Venue, Community survey

## Abstract

**Background:**

Harmful gambling has been identified as an important public health issue that affects individuals, families and the broader community. One gambling product, electronic gambling machines (EGMs), has been associated with significant gambling harm in Australia. There has been limited research that has explored community perceptions of EGMs and attitudes towards reform. This study, conducted in NSW, Australia, aimed to explore community use of EGM venues (clubs and hotels containing EGMs), attitudes towards EGMs and whether the use of these venues influenced attitudes towards EGM reform.

**Methods:**

An online survey was conducted with 500 adults aged 16 years and over, representative of the population for age and gender. Discrete choice and open-ended questions were used to gather data on gambling behaviours, use of and attitudes towards EGMs and EGM venues and support for gambling harm reduction measures.

**Results:**

Three quarters of participants had visited an EGM venue in the previous year. Participants who had attended such venues were significantly more likely to use EGMs at least once per month. Participants attended EGM venues for a range of reasons including use of non-gambling facilities such as restaurants, the social aspects of the venue and ease of access to the venue. Some participants also attended EGM venues specifically for the gambling facilities. Most participants identified some negative impacts of EGMs for local communities and were supportive of measures to reduce the number of EGMs and prevent children's exposure to EGMs in such venues.

**Conclusions:**

This study shows a high level of support for EGM reform amongst both individuals who attend EGM venues and also those who do not. There is potential for government to further regulate EGMs and the environments where they are located.

## Background

Electronic gambling machines (EGMs, also known as “pokies” or poker machines) have been identified as the gambling product that causes the most harm to individuals and communities in Australia [1]. While the number of people using EGMs has decreased [2], the amount of money lost on EGMs has stayed largely the same over the last 10 years [3]. Over $12 billion was lost on EGMs in 2015/2016, a significant proportion of the $23.6 billion lost on all forms of gambling during the same time period [3]. The Australian Productivity Commission [2010] stated that (a) an estimated 40% of the total share of Australian gaming machine losses come from problem gamblers, (b) the majority of EGM revenue was from individuals who used EGMs weekly or more and (c) the increased use of EGMs was associated with increased risk of gambling harm [4].

In Australia, most EGMs are based in community clubs and hotels, with EGMs in these venues generating the majority of gambling revenue (55%) in Australia [4]. In 2016, there were 195,631 EGMs in Australia, with just under half of these (94,408 machines) in New South Wales (NSW) [3]. Research shows that per capita expenditure on EGMs in NSW is much higher ($1022) than in other Australian states such as Queensland ($616) and Victoria ($558) [3], although figures do not include EGM expenditure from casinos and are not able to be compared to Western Australia where there are no EGMs outside its sole casino.

Researchers have identified a range of specific risk factors associated with the presence of EGMs in communities. The first is that EGM's, and higher EGM losses, were concentrated in areas with higher levels of disadvantage [5]. For example, data suggests that in 2016, $800 million was lost on EGMs in the NSW suburb of Fairfield, an area with a very high level of social disadvantage and deprivation [6]. This raises significant concerns about the impacts of EGMs on social and health inequity. For example, EGMs have been linked to a number of health and social issues, with recent research suggesting that areas with higher numbers of EGMs also reported high rates of family violence [7]. Second are risk factors associated with accessibility of EGMs in community settings [8]. Studies have demonstrated that proximity to gambling venues is linked to individual gambling behviours [9] and that those who live closer to venues spend more money annually on EGMs [10]. Third is the co-location of gambling with the consumption of other risky activities in these venues, such as alcohol. In a study by Deans and colleagues, young men stated that local hotels were “a hub for different forms of gambling” (p. 115), whereby multiple gambling options, peer influences and alcohol consumption contributed to risky gambling behaviours [11]. The final factor is the exposure of children to EGMs in community settings. Research has demonstrated that while children are not legally allowed onto gaming room floors, those who attended venues had visible and audible exposure to EGMs, and were able to describe the characteristics of EGMs [12]. Some had positive perceptions of EGMs, with children who regularly attended venues saying that they wanted to gamble on EGMs when they were older [12].

What is less clear is the range of socio-cultural and industry-related factors that influence attendance at venues and, subsequently, shape community perceptions of the risks and benefits associated with gambling venues. To date, there has been limited research exploring the broad range of factors that may encourage or create pathways for individuals into community-based EGM venues. A small amount of research suggests that increased gambling participation and expenditure may be linked to the extent to which gambling venues are seen as safe entertainment settings [13, 14]. Researchers have also identified the range of promotional factors that may shape attitudes towards gambling venues, indicating that in the absence of an ability to directly promote EGMs, venues focus on the promotion of family-friendly activities, cheap meals and other forms of entertainment to encourage individuals into venues [15].

Research from other areas of public health, such as drug-related harm, suggests that harms are a product of the environments of individuals [16]. Environments are influenced by a range of micro and macro risks that extend beyond the individual and encompass physical, social, economic and policy environments [17]. In order to develop harm reduction strategies in gambling, it is important to understand the broader range of structural and environmental factors that may contribute to gambling risks in communities, including (a) community perceptions of the risks and benefits of EGM venues [18, 19] and (b) their support for strategies that may reduce the risks associated with these venues [20]. The current study aimed to understand the attitudes of a sample of community members in NSW, Australia, including the behaviours of individuals who attended EGM venues (clubs and hotels), and in particular how frequency of attendance at venues influenced attitudes and support for harm prevention measures associated with EGMs and venues. The research was guided by five questions:

1. What factors influence attendance at EGM venues?
2. Are individuals who attend EGM venues more likely to use EGMs than those who do not?
3. How do community members perceive EGMs in their community?
4. Does the community support increased regulations to reduce the harms associated with EGM venues?
5. Are there differences in attitudes towards EGMs and EGM reform between people who visit EGM venues and those who do not?

## Methods

### Approach

The study used data from an online survey of 500 individuals aged 16–82 years who were residents of NSW, Australia. This research received ethics approval from the Deakin University Human Research Ethics Committee.

### Setting

We chose to focus this study on NSW for four reasons. First, NSW was the first state in Australia to introduce EGMs into community clubs, with clubs becoming increasingly reliant on the revenue from EGMs [21]. Second, NSW has the highest number of EGMs in Australia and the highest per capita losses on EGMs [2]. Third, there has been limited transparency around losses on EGMs in NSW [22], despite this being the practice of governments in other states such as Victoria [23]. Finally, while research since the 1990s has demonstrated significant community support for gambling reform (for a discussion, see Thomas and colleagues [20]), no research has specifically looked at the relationship between use of EGM venues and attitudes towards community-based reform in NSW.

### Recruitment and sampling

Data were collected in May 2017 using an online panel company. Quotas were set to ensure the sample was representative of the NSW population for age and gender [24]. Participants aged 16 and 17 years were included because individuals younger than 18 may attend community gambling venues that contain EGMs, and research suggests that children begin to think about gambling on EGMs prior to turning 18 years old [12]. Panel members who were eligible for the study were sent an invitation to complete the survey. On completion of the survey, participants were reimbursed with points that could then be redeemed for gift vouchers online. Participants were excluded if they were younger than 16 years, if they did not give consent, if the age and gender quota was full or if they did not complete the full survey. Participants were also excluded if answers were nonsensical or contradictory.

### Data collection

The survey was hosted through Qualtrics survey software. First, data were collected on a range of socio-demographic factors that included age, gender, postcode, education level, employment and whether they had children. Second, we collected information about gambling behaviour, including frequency and use of gambling products (casino gambling, EGMs, horse betting, sports betting and other), and gambling status (the Problem Gambling Severity Index (PGSI) was used as a measure of problem gambling status [25]). Third, we asked participants about their attendance at clubs or hotels that contained EGMs, referred to in this paper as EGM venues. Participants were asked to qualitatively describe the reasons they attended these venues. Open-ended questions were used to explore perceptions of the risks and benefits of EGMs for their local community. Finally, participants were asked to indicate on a 4-point scale the extent to which they agreed or disagreed with a range of statements relating to EGM reform. Given research which suggests that children are exposed to EGMs in community venues [12], we were particularly interested in exploring whether there was community support for the separation of EGMs and children’s areas in EGM venues. We were also interested in the extent to which the community supported broad harm prevention strategies relating to EGMs in EGM venues.

### Data analysis

Data were downloaded to SPSS 22.0 for checking and cleaning. Postcodes were used to determine socio-economic status of the area of residence (using the Socio-Economic Indexes for Areas, Index of Relative Socio-economic Disadvantage) [26], and descriptive analysis on demographic variables produced the sample description.

Responses from PGSI questions were calculated to categorise participants’ gambling status as non-problem gambling (score 0), low-risk gambling (scores 1–3), moderate risk gambling (scores 4–7) and problem gambling (scores of 8 or above). Individuals who reported not gambling in the previous 12 months were “non-gamblers”. Ten self-reported non-gamblers received PGSI scores above zero (indicating some level of gambling behaviour and risk); these individuals were re-classified from the “non-gambling” group into the appropriate category given their PGSI score.

To test for differences between individuals according to the frequency of attendance at EGM venues, the sample was split into three groups: did not attend EGM venues, attended infrequently (less than once a month) and attended frequently (at least once a month). Chi-squared (*χ*^2^) tests of association were used to test for significant differences between groups at the 95% level.

Qualitative data were analysed using a constant comparative method [27]. The first two authors read and re-read responses to develop preliminary themes, which were compared according to socio-demographic factors, venue use and gambling behaviours. These were then discussed with the broader team to determine how they fit with the data as a whole. In presenting the data, minor typographical errors in the qualitative responses provided by the participants were corrected. Any capitalisation or emphasis by participants was not changed.

## Results

### Sample description

Table 1 provides the socio-demographic and gambling characteristics of the sample. Participants ranged from 16 to 82 years, with a mean age of 45.15 years (SD 17.6). The sample contained 20 individuals aged 16 and 17 years old, the majority (*n* = 13) of whom were male. Most participants (*n* = 384, 76.8%) were from middle or high socio-economic areas, had an education level above year 12 (*n* = 364, 72.8%), and were employed in full-time, part-time or casual work (*n* = 295, 59.0%). Just under one third of the sample (*n* = 151, 30.2%) had a child under 18 years at the time of data collection. Just under 40% (*n* = 199, 39.8%) had experienced some level of gambling-related harm (PGSI score of 1 or more), with 84 participants (16.8%) reporting problem gambling behaviours (PGSI score of 8 or more). Six participants under 18 years old reported some level of gambling-related harm (PGSI score of 1 or more); of which three were moderate risk gamblers and another three were problem gamblers.

**Table 1:** Socio-demographic and gambling characteristics of the sample

Characteristic		Number	Percent
Age	16–17 18–24 25–34 35–44 45–54 55–64 65 or older	20 60 90 83 79 71 97	4.0 12.0 18.0 16.6 15.8 14.2 19.4
Gender	Male Female	246 254	49.2 50.8
Socio-economic status	Low (1–3) Middle (4–7) High (8–10)	116 194 190	23.2 38.8 38.0
Education	Year 12 or less Cert I, II, III, IV Diploma/advanced Bachelor’s degree Graduate diploma/certificate Postgraduate	136 62 73 144 24 61	27.2 12.4 14.6 28.8 4.8 12.2
Employment status	Working full-time Working part-time/casually Unemployed but looking for work Homemaker Retired Full-time student Other	184 111 16 33 108 38 10	36.8 22.2 3.2 6.6 21.6 7.6 2.0
Children	No children under 18 years At least one child under 18 years	349 151	69.8 30.2
Problem gambling status	Non-gambling Non-problem gambling Low-risk gambling Moderate-risk gambling Problem gambling	102 199 67 48 84	20.4 39.8 13.4 9.6 16.8

Table 2 presents the frequency of participation in gambling. Just over three quarters of the sample (*n* = 388, 77.6%) reported having participated in some form of gambling in the previous 12 months. This included one third of participants aged under 18 years (*n* = 7, 35.0%). Over half of participants had gambled on EGMs in the previous year (*n* = 260, 52.0%), with fewer participants gambling on horse betting (*n* = 228, 45.6%), casino gambling (*n* = 177, 35.4%) and sports betting (*n* = 171, 34.2%). Two thirds (*n* = 330, 66.0%) of participants reported that they gambled on other forms of gambling at least once a month, including lotteries, buying scratch tickets (scratchies), Keno, raffles, bingo or dog racing. There was an association between frequent EGM use and experience of gambling- related harm. Participants who gambled on EGMs at least monthly were more likely to score as moderate risk gamblers or problem gamblers on the PGSI [χ² = 6.01, *p* = 0.014].

**Table 2:** Frequency of participation in different types of gambling

Type of gambling	Never	Less than once a month	Monthly or more	Total use
	*n* (%)	*n* (%)	*n* (%)	*n* (%)
Gambling on EGMs	240 (48.0)	149 (29.8)	111 (22.2)	260 (52.0)
Betting on horses	272 (54.4)	134 (26.8)	94 (18.8)	228 (45.6)
Gambling at the casino	323 (64.6)	116 (23.2)	61 (12.2)	177 (35.4)
Betting on sports	329 (65.8)	70 (14.0)	101 (20.2)	171 (34.2)
Other gambling	170 (34.0)	138 (27.6)	192 (38.4)	330 (66.0)

Over three quarters of participants reported attending an EGM venue in the previous 12 months (*n* = 395, 79.0%), with over one third of participants attending at least monthly (*n* = 181, 36.2%). Over half (*n* = 232, 58.7%) of the participants who attended EGM venues had gambled on EGMs in the previous 12 months. Partcipants who attended an EGM venue at least once a month were significantly more likely to report EGM use in the previous year (*n *= 123, 68.0% of those who attended at least monthly), compared to those who went to an EGM venue less frequently [χ² = 28.94, *p* < 0.001]. Participants who visited EGM venues in the previous 12 months were also more likely to bet on horses at least once a month (*n *= 86, 91.5% of those who bet on horses monthly or more) [χ² = 10.88, *p *= 0.001].  

### Participation in activities at EGM venues

Qualitative responses were used to document the reasons why participants visited EGM venues. It is important to note that of the 17 participants in the survey who gave no response to this question, 11 were categorised as problem gamblers.

The vast majority of participants who attended EGM venues stated that they attended for non-gambling activities (*n* = 390, 98.7%). These activities were grouped into four categories. First was the use of the restaurant or bar (*n* = 154, 39.0%). Many participants referred to the value for money or affordability of the restaurants. References to the consumption of alcohol at the venue were often combined with descriptions of activities such as socialising with friends or with a meal. Second were social reasons, such as meeting up with friends or family or to socialise (*n* = 100, 25.3%). For example, one male aged 41 who was a non-gambler said he attended to “socialise with friends over a couple of drinks”. Some (*n* = 63, 15.9%) also described the “good atmosphere”, including that it was “fun”, “relaxing” or “comfortable”. Third were those who visited the venue because it was accessible, local and easy to get to (*n* = 74, 18.7%). Fourth were non-gambling entertainment activities including seeing live bands, shows, watching sport, regular club member events such as trivia nights or members’ draws or to use the sporting facilities (*n* = 39, 9.9%).

Some participants stated that while they attended EGM venues, it was not their choice to do so (*n* = 29, 7.3%). For these participants, the decision to attend had been made by others, including family and friends, or the venue had been chosen for sporting team events or functions. Some stated that while they would prefer not to attend these venues, there were no other alternatives in their local area. For example, one male commented on the lack of options in his area:

That’s where we meet. Besides where else is there to go?—Male, 64 years, attended frequently, non-problem gambler.

A minority of participants stated that their reason for attending the venue was for gambling (*n* = 16, 4.1%). These people were often at high risk of gambling-related harm according to their PGSI score. For example, one female aged 38 years who was classified as a problem gambler said her attendance at the venue was because “they have a lot of machines”.

### Community attitudes towards EGMs

When asked to describe positive and negative aspects of EGMs for their local community, just over one fifth of participants (*n* = 109, 21.8%) provided both negative and positive responses towards EGMs, just under half of participants (*n* = 214, 42.8%) provided a negative response only and one in ten (*n* = 52, 10.4%) provided a positive response. A quarter of participants (*n* = 125, 25.0%) provided neither a negative or positive response to this question, for example “I don’t know” or provided a response that could not be categorised as either positive or negative. The following provides results for the participants who provided a distinct negative or positive response towards EGMs.

Just under two thirds of participants qualitatively described at least one negative impact of having EGMs in their local community (*n* = 323, 64.6%). For example, participants described negative financial consequences (*n* = 105, 32.3%), the role of EGMs in gambling addiction (*n* = 107, 33.1%) and negative social impacts on communities (*n* = 65, 20.1%). While there were a range of specific negative social impacts identified, such as crime and mental health issues, just under half of participants who identified these impacts described the negative impact of EGMs on families and children (*n* = 29). Participants described families being impacted as a result of another individual’s gambling, for example, family stress, family financial problems and divorce or family conflict. For example, one female stated that EGMs led to the:

Destruction of families due to addictive behaviour.—Female, 74 years, does not attend venues, non-gambler.

A small number of participants (*n* = 5), all under 40 years old, described the effect of EGMs on children, including its effect on children’s future gambling behaviours. For example, one 18-year-old female described the impact of increased accessibility on exposing children to EGMs:

Proximity to home means younger people are more likely to be exposed to it.—Female, 18 years, attended venues infrequently, non-gambler.

Others commented on the extent to which EGMs were available and accessible in the community (*n* = 29, 9.0%). For example, one participant stated EGMs were “dangerously accessible”, while another said, “they are everywhere, WHY?” Others stated that accessibility had a direct link with increased harm from EGMs, “it makes it easier to gamble if they are within reach”.

Around one third of participants described that there were some benefits associated with EGMs (*n* = 161, 32.2%). This included 78 participants who attended EGM venues at least once a month who provided a positive response. Some participants (*n* = 38) described the positive benefits of employment and that profits were used to fund community projects, charities and activities, taxes and community-based sports:

Positive side profits, particularly from local service and community clubs, help many junior sports, charities and local community institutions (hospitals, schools, age care, etc.) who without the funds would not provide many of the services in their local community.—Male, 65 years, attended venues frequently, low-risk gambler.

Others (*n* = 65) viewed EGMs as a positive form of entertainment in the community that created social connections. For example, one young woman stated that EGMs contributed to positive social interactions, but also that the winnings from EGMs would help hotels make money:

[EGMs] bring people together by cheering and excitable moments of winning. Buying more drinks from the pubs and them making more money.—Female, 22 years, attended venues infrequently, moderate-risk gambler.

Some of those who believed that EGMs were positive for communities had caveats on this, for example, that EGMs were only fun for people who were responsible or in control.

It’s not good for the weak people who don’t know when to stop but for others it’s a lot of fun.—Male, 24 years, attended venues frequently, problem gambler.

### Support for EGM harm reduction and prevention measures

Table 3 provides information about perceptions of the reliance of venues on EGM revenue, by their frequency of attendance at these venues. Over 90% of participants agreed or strongly agreed (*n* = 454, 90.8%) that clubs and hotels should be less reliant on money from EGMs. However, individuals who attended EGM venues more frequently were significantly less likely to strongly agree with this statement compared to other groups [*χ*^2^ = 15.81, *p* = 0.015].

**Table 3:** Attitudes towards EGMs by the frequency of attendance at venues

		Venue attendance				Sig	
		Did not attend 105 (21.0%)	Attended infrequently 214 (42.8%)	Attended frequently 181 (36.2%)	Total 500 (100.0%)		
						*χ* ^2^	*p* value
It would be better if clubs and hotels were not so reliant on money from EGMs	Strongly agree	47 (44.8%)	89 (41.6%)	59 (32.6%)	195 (39.0%)	15.81	.015*
	Agree	49 (46.7%)	115 (53.7%)	95 (52.5%)	259 (51.8%)		
	Disagree	8 (7.6%)	10 (4.7%)	25 (13.8%)	43 (8.6%)		
	Strongly disagree	1 (1.0%)	0	2 (1.1%)	3 (0.6%)		
I would prefer to attend a club or hotel that did not have EGMs	Strongly agree	39 (37.1%)	54 (25.2%)	34 (18.8%)	127 (25.4%)	24.87	< .001*
	Agree	43 (41.0%)	99 (46.3%)	68 (37.6%)	210 (42.0%)		
	Disagree	17 (16.2%)	54 (25.2%)	70 (38.7%)	141 (28.2%)		
	Strongly disagree	6 (5.7%)	7 (3.3%)	9 (5.0%)	22 (4.4%)		

*Indicates values significant at the 95% level

While around two thirds of participants agreed or strongly agreed that they would rather attend a venue that did not have EGMs (*n* = 337, 67.4%), there were significant differences according to the level of attendance [*χ*^2^ = 24.87, *p* < 0.001]. Those who attended EGM venues frequently were more likely to disagree with this statement, with just under half (*n* = 79, 43.6%) disagreeing or strongly disagreeing.

Support for other harm reduction measures can be found in Table 4. Most people agreed or strongly agreed that the NSW government should increase the regulation of EGMs (*n* = 407, 81.4%), including reducing the number of EGMs in NSW (*n* = 384, 76.8%). Although two thirds of the sample supported the removal of EGMs from local communities (*n* = 330, 66.0%), participants who attended EGM venues frequently were more likely to disagree with this statement (44.2% compared to 23.8% of people who do not attend venues).

**Table 4:** Attitudes towards EGM harm reduction and prevention measures by the frequency of attendance at venues

		Venue attendance				Sig	
		Did not attend 105 (21.0%)	Attended infrequently 214 (42.8%)	Attended frequently 181 (36.2%)	Total 500 (100.0%)		
						*χ* ^2^	*p* value
EGM harm reduction and prevention measures							
The NSW government should increase regulation of EGMs	Strongly agree	45 (42.9%)	84 (39.3%)	59 (32.6%)	188 (37.6%)	10.06	.122
	Agree	42 (40.0%)	99 (46.3%)	78 (43.1%)	219 (43.8%)		
	Disagree	14 (13.3%)	27 (12.6%)	40 (22.1%)	81 (16.2%)		
	Strongly disagree	4 (3.8%)	4 (1.9%)	4 (2.2%)	12 (2.4%)		
The number of EGMs in NSW should be reduced	Strongly agree	44 (41.9%)	67 (31.3%)	50 (27.6%)	161 (32.2%)	17.08	.009*
	Agree	39 (37.1%)	107 (50.0%)	77 (42.5%)	223 (44.6%)		
	Disagree	17 (16.2%)	38 (17.8%)	49 (27.1%)	104 (20.8%)		
	Strongly disagree	5 (4.8%)	2 (0.9%)	5 (2.8%)	12 (2.4%)		
I would support the removal of EGMs from my local community	Strongly agree	36 (34.3%)	57 (26.6%)	45 (24.9%)	138 (27.6%)	16.43	.012*
	Agree	44 (41.9%)	92 (43.0%)	56 (30.9%)	192 (38.4%)		
	Disagree	20 (19.0%)	54 (25.2%)	63 (34.8%)	137 (27.4%)		
	Strongly disagree	5 (4.8%)	11 (5.1%)	17 (9.4%)	33 (6.6%)		
Measures that reduce children’s exposure to EGMs							
Children should not be able to see or hear EGMs in clubs and hotels	Strongly agree	50 (47.6%)	100 (46.7%)	82 (45.3%)	232 (46.4%)	3.84	.699
	Agree	40 (38.1%)	92 (43.0%)	80 (44.2%)	212 (42.4%)		
	Disagree	14 (13.3%)	20 (9.3%)	15 (8.3%)	49 (9.8%)		
	Strongly disagree	1 (1.0%)	2 (0.9%)	4 (2.2%)	7 (1.4%)		
There should be greater separation between gambling products and family areas in clubs and hotels	Strongly agree	50 (47.6%)	87 (40.7%)	73 (40.3%)	210 (42.0%)	8.68	.192
	Agree	41 (39.0%)	110 (51.4%)	80 (44.2%)	231 (46.2%)		
	Disagree	12 (11.4%)	15 (7.0%)	25 (13.8%)	52 (10.4%)		
	Strongly disagree	2 (1.9%)	2 (0.9%)	3 (1.7%)	7 (1.4%)		
Clubs and hotels can be “family friendly” and also contain EGMs	Strongly agree	10 (9.5%)	24 (11.2%)	35 (19.3%)	69 (13.8%)	27.67	*<* .001*
	Agree	48 (45.7%)	124 (57.9%)	101 (55.8%)	273 (54.6%)		
	Disagree	29 (27.6%)	57 (26.6%)	32 (17.7%)	118 (23.6%)		
	Strongly disagree	18 (17.1%)	9 (4.2%)	13 (7.2%)	40 (8.0%)		

*Indicates values significant at the 95% level

Almost 90% of participants agreed that children should not be able to see or hear EGMs in venues (*n* = 444, 88.8%) and that there should be greater separation between gambling products and family areas in venues (*n* = 441, 88.2%). Just over two thirds agreed that venues could be family friendly and also contain EGMs (*n* = 342, 68.4%). Again, participants who attended venues frequently were more likely to agree with this statement [*χ*^2^ = 27.67, *p* < 0.001].

## Discussion

This study aimed to explore community attitudes and behaviours in relation to EGM venues, the factors that influence attendance, and levels of support for increased regulation to reduce the harms associated with EGM venues. The results raise three key areas for discussion.

First, many people in this sample attended EGM venues primarily for non-gambling activities, such as affordable meals and the accessibility of the venue. However, participants who visited more frequently also gambled on EGMs more often. This suggests that although participation in gambling is not the primary reason for visiting, the presence of EGMs in these venues may encourage a pathway to participation in this form of gambling. Further research is required to explore the pathways by which individuals may transition from being non-gamblers to gambling frequently within venues and which strategies may disrupt pathways to EGM use. While EGM venues provide some beneficial services through community funding, evidence shows that these benefits are outweighed by the harms associated with gambling [28]. Researchers should also explore whether non-gambling activities within venues create perceptions of lesser risks associated with the gambling products there.

Second, participants identified a range of positive and negative factors associated with EGMs being located within their communities. While the majority of participants recognised that EGMs had at least some negative consequences for communities, one fifth of participants said they used EGMs at least once a month. This is consistent with a similar study conducted in Victoria by the research team, which found that although participants perceived EGMs as harmful, just over half reported using EGMs in the previous 12 months [20]. This may suggest that there are broader socio-cultural and environmental factors that influence individual participation in EGMs at community venues. This study has also found that for some individuals who perceived there were positive associations with EGMs, these were focused on the community benefits of EGMs. Public health programs should seek to educate communities about any discrepancy between community contributions and harms to ensure that individuals have an informed perception of EGMs.

Finally, the majority of participants were supportive of EGM harm reduction and prevention measures. Strong support was evident for any changes that restricted children’s exposure to EGMs, including amongst individuals who frequently attended EGM venues. The community attitudes revealed in this study suggest that the community may also be supportive of a reduction in the number of EGMs in NSW. This finding is consistent with other Australian gambling surveys that have shown support for gambling policy change [20, 29–31]. However, despite this, there has been limited reduction in numbers of EGMs in Australia. For example, in 2017, the Victorian government announced it would keep the numbers of EGMs at the same level for the next 25 years [32], while NSW has seen a decrease of less than 4500 EGMs in the past 10 years [2]. While community support for EGM reform is a potentially important voice in public debate, those concerned with reducing harms from gambling need to frame their messages carefully and anticipate a response from interests such as EGM venues, manufacturers and governments, all of whom reap benefits from EGM revenue. Based on prior experience [33], it is likely that any attempt to change EGM legislation will face significant opposition from the gambling industry, including sophisticated campaigns to shift community attitudes. Building coalitions, raising the public profile of key issues, educating the community about the imbalance between harms and benefits [34] and arguing for product regulation [34, 35] are likely to assist those concerned with reducing gambling harms to respond to such resistance. These strategies have been effective in the Australian state of Tasmania, with some recent policy commitments from political parties supporting the removal of EGMs from the community back to casino-based settings [36, 37]. Although effective harm reduction measures may result in a reduction of EGM revenue [3], governments should view this in the context of the social and economic benefits resulting from regulatory changes, in line with the attitudes of communities that support EGM reform.

This study has several limitations. Although the study sampled for age and gender according to the NSW population, participants reported higher levels of gambling and had higher risk gambling status (PGSI scores) compared to those in the previous telephone-based prevalence surveys [4, 38]; however, this finding is consistent with other online gambling studies [39]. While it is important to explore the perspectives of people who have experienced gambling harm, the views of participants in this study, with high rates of gambling harm, may not be generalisable to the general population. This study aimed to provide an overview of community attitudes towards EGMs and EGM reform, and although we have not tested strategies that may enable venues to transition away from their reliance on EGM revenue, further research should explore this avenue in more detail. It is essential that such research be conducted independent of any interests that may be conflicted due to any reliance on EGM revenue. Finally, while findings of this study suggest strong community support for regulation of EGMs, it should also be noted that the study was conducted in the absence of any industry lobbying to counter such measures, and so the extent to which levels of community support might be eroded by effective industry campaigning cannot be known. It is therefore important to regularly monitor public opinion towards EGM reform, as well as the public relations and lobbying activities of the gambling industry, and any publicity surrounding this issue.

## Conclusion

The findings of this study highlight that the majority of a sample of community members in NSW, including those who attend EGM venues, recognise that EGMs have at least some negative consequences for communities and support measures to reduce EGM harm. There is potential for governments to further regulate EGMs and the venues where they are located, to significantly prevent and reduce gambling harm in Australian communities.

## Abbreviations

EGM: Electronic gambling machine
NSW: New South Wales
PGSI: Problem Gambling Severity Index
